# A gamified augmented reality vocational training program for adults with intellectual and developmental disabilities: A pilot study on acceptability and effectiveness

**DOI:** 10.3389/fpsyt.2022.966080

**Published:** 2022-08-04

**Authors:** Bhing-Leet Tan, Frank Yunqing Guan, Ivy Mun Wah Leung, Sharon Yi-May Kee, Oran Zane Devilly, Alice Medalia

**Affiliations:** ^1^Health and Social Sciences Cluster, Singapore Institute of Technology, Singapore, Singapore; ^2^Occupational Therapy Department, Institute of Mental Health, Singapore, Singapore; ^3^Infocomm Technology Cluster, Singapore Institute of Technology, Singapore, Singapore; ^4^Department of Psychiatry, New York State Psychiatric Institute, Columbia University Vagelos College of Physicians and Surgeons, New York, NY, United States

**Keywords:** augmented reality, vocational training, transfer of learning, cues and prompts, intellectual and developmental disabilities, Neuropsychological and Educational Approach to Remediation

## Abstract

**Objectives:**

The Augmented Reality Games to Enhance Vocational Ability of Patients (REAP) was an augmented reality vocational training program that provided skills training in the context of a psychiatric rehabilitation program. It was implemented over 10 weeks and consisted of gamified augmented reality café training scenarios and bridging group activities to facilitate transfer of learning to the work context. This pilot study aimed to explore the acceptability and effectiveness of the REAP program when carried out with adults with intellectual and developmental disabilities attending work therapy. Its objectives were: (1) to obtain feedback from participants and trainers on their experiences and acceptability of the REAP program and (2) to measure changes in vocational and cognitive skills of participants in the REAP program.

**Materials and methods:**

This was a pretest–posttest mixed methods study. 15 adults with intellectual and developmental disabilities attending work therapy in a non-profit organization participated in the REAP program and their vocational trainers were involved in assisting in this program. Feasibility Evaluation Checklist (FEC) and the Neurobehavioral Cognitive Status Exam (Cognistat) were administered at baseline, post-training and eight weeks after training. The participants and their trainers also provided user feedback *via* semi-structured interviews.

**Results:**

Majority of the participants and trainers found the REAP program to be useful and interesting. They also found that the augmented reality games were user-friendly and provided a unique opportunity to acquire new skills. Participants who engaged in this program showed a significant improvement in vocational skills and aspects of cognitive skills, which were maintained eight weeks after training.

**Conclusion:**

The gamified augmented reality vocational training was feasible and accepted by both adults with intellectual and developmental disabilities and their trainers. When integrated with bridging sessions to facilitate transfer of learning to existing work therapy, participants on the REAP program showed significant improvements in vocational skills and aspects of cognitive skills. Future experimental studies with larger sample size could provide stronger evidence on its effectiveness in improving vocational outcomes.

## Introduction

Adults with intellectual and developmental disabilities can lead meaningful and fulfilling lives, when interventions are in place to equip them with skills or adaptive strategies to maximize participation in daily living, leisure, community living and work. Technology and assistive devices have traditionally been used to improve functional outcomes through skills training or task adaptations. For example, a computer game was used to train decision-making skills in a group of adults with intellectual disabilities (1). Another study explored the use of vibrating watches as a time management tool to assist in task transition and completion (2). Recently, a mindfulness and relaxation game was designed to teach stress management strategies to persons with intellectual disabilities (3). Skills training using a carefully designed gamified platform has the ability to facilitate the learning of new tasks in a scaffolded and interesting manner, thus promoting internal motivation to engage in the learning process (4).

While computer-based skills training packages allow persons with intellectual and developmental disabilities to learn in a structured manner, recent advancements in augmented and virtual reality have enabled rehabilitation practitioners to provide a more immersive training environment. Virtual Reality (VR) transports the users to a fully immersive environment, which replicates aspects of reality and enables stimulation of the senses as well as interaction with objects in a simulated environment (5). On the other hand, Augmented Reality (AR) enables the users to perceive a more realistic training environment, as the real environment is superimposed on virtual three-dimensional graphics and images (6). Therefore, users will still be able to view the real environment while they interact with the virtual objects. As a result, AR has the added advantage of providing users with better control of their actions and balance, thus minimizing hazards such as colliding with walls (7).

Despite these beneficial features, the use of AR as a skills training platform for adults with intellectual and developmental disabilities has not been extensive. In an application of AR to adults with autism, a preliminary study considered the use of AR smartglasses as a social communication aid (8). Other studies explored the use of AR in daily living or community living skills training for adults with intellectual disabilities. One study used video models to teach the step of ironing, making bed and setting an alarm clock (9). These video models were activated by the rear-facing camera on iPads and appeared as an overlay across a target spot. Two studies used mobile applications that combined AR features with global positioning systems (GPS), to provide real-time navigation cues to participants (10, 11). One of the mobile applications could also function as a search engine for location-based information from the selected venue (11). Gamification using an AR application was also implemented to teach the use of an automated teller machine (ATM), where the AR mobile phone application provided cues over an ATM simulator presented on an iPad (12). Overall, these studies were small sample designs involving not more than five research participants.

In the functional area of work, attempts have been made to utilize AR to train various vocational skills, in order to enhance employability and vocational opportunities. One study used an AR platform to present video instructions on horticulture work for eight adults with intellectual disabilities (13). These contents were activated at selected GPS coordinates and displayed on tablets. Another team developed a marker-based mobile AR application named “Paint-cAR,” to support participants in a car maintenance vocational rehabilitation program who were learning how to repair car paints (14). Results of this cross-sectional evaluation study showed that participants felt the AR training boosted their confidence and satisfaction in picking up this vocational skill. Lastly, AR was also used as a vocational task prompting system called ARCoach, where three participants learning how to assemble food items were given AR-generated audio and visual cues whenever they made a mistake in the tasks (6).

Despite wanting to work, adults with intellectual and developmental disabilities often have difficulties obtaining and sustaining employment (15, 16). Services for adults with intellectual and developmental disabilities offer a variety of vocational rehabilitation programs, ranging from work skills training, sheltered employment, supported employment to hybrid models of employment (15, 17). In order to maximize their chances of attaining supported or open employment, skills training programs are often implemented to prepare them for a variety of jobs, in areas such as food and beverage, retail, housekeeping, etc. Simulating different job tasks of varying complexities in the natural environment can be challenging and time-consuming, which will limit the number of clients who can be trained within a specific period. In addition, it is assumed that repeated practice of essential steps of work tasks will result in eventual work competence. However, open employment often involves situations that require problem solving and responding to social cues, which may be difficult to enact in real-life training (18). Such situations may include knowing how to respond when a customer accidentally knocks over another customer’s drinks, knowing what to do when the towel near the kitchen stove catches fire, etc. Gamification using AR may be a viable solution in providing a systematic and enjoyable way of training vocational skills, which allow simulation of problematic work scenarios without the need for huge training spaces. Therefore, there are possible benefits in using AR to maximize clients’ potential for higher vocational attainment. However, as described earlier, research on the use of AR in vocational rehabilitation is still at the infancy stage. Hence, there is a need to investigate the acceptability, feasibility and effectiveness of using AR in improving vocational skills.

The Augmented Reality Games to Enhance Vocational Ability of Patients (REAP) was an AR-enabled vocational training program that provided adjunctive skills training in the context of an existing vocational rehabilitation program. A User-Centered Design approach was adopted in the AR prototype development, which emphasized on the clients’ needs and goals to ensure meaningful gamification (19). Taking this into consideration, the program used the framework from the Neuropsychological and Educational Approach to Remediation (NEAR). NEAR is derived from neuropsychology, educational psychology, behavior learning theory and theory of self-determination (20). It is a cognitive rehabilitation framework and program that incorporates education psychology’s emphasis on creating a learning environment that enhances motivation to learn. Therefore, features such as personalizing characters, contextualizing the games to simulate real work environment, provision of user choices, evoking interest through attractive multi-media images were incorporated into the gamified training scenarios in REAP. As important, the AR games were built to provide attributional and strategy feedback, so that trainers could guide clients to evaluate their task responses (21). The storyboard for each training scenario was developed based on the framework of Perceive, Recall, Plan and Perform system of task analysis, to break down the work tasks into steps to scaffold learning (22, 23). In addition to using the NEAR framework to guide motivational enhancements, the AR games used NEAR-style bridging activity groups to facilitate transfer of learning from the games to the existing vocational rehabilitation programs and other aspects of daily lives. Even though adults with intellectual and developmental disabilities have cognitive limitations, REAP attempted to harness the therapeutic components of rehearsal and strategy-building to facilitate vocational skills acquisition and functional gains.

The aim of this pilot study was to explore the acceptability and effectiveness of the REAP program. Its objectives were:

1. To obtain feedback from participants and trainers on their experiences and acceptability of the REAP program.
2. To measure changes in vocational and cognitive skills of participants in the REAP program.

## Materials and methods

### Study design

This was a pilot study to evaluate REAP’s augmented reality (AR) gamified platform prototype and training sessions, to ascertain the feasibility, acceptability, safety, and effectiveness for adults with intellectual and developmental disabilities. A pretest–posttest mixed methods design was adopted, with follow-up after eight weeks. Quantitative data such as vocational skills and cognitive functions was collected at three time points. A user feedback interview was also conducted with the research participants and the trainers, yielding quantitative and qualitative data.

### Participants and setting

The study was conducted in collaboration with Bizlink Center, a non-profit organization with the mission of assisting persons with disabilities in the provision of employment through vocational training and various employment programs. The pilot trial was conducted in Bizlink Headquarters, which provided work therapy in their workshops and social enterprises, as well as their Day Activity Center, which provided aspects of work training. Clients who met the following inclusion and exclusion criteria were recruited for this pilot study.

Inclusion criteria:

• Persons with intellectual or developmental disabilities who were receiving services at Bizlink.
• Able to converse in English and understand English instructions, as the AR games were in the English medium.
• Able to ambulate without any physical assistance.

Exclusion criteria:

• Unable to speak and understand English.
• Co-morbid epilepsy, which would affect gains from AR vocational training.

As the study was conducted during the COVID-19 pandemic period, open or supported employment were not the main goals for the majority of Bizlink’s clients and their caregivers. Nevertheless, the center strived to maintain meaningful engagement of work tasks and vocational opportunities that matched the clients’ interests and functional performance.

Besides the clients, user feedback interviews were also conducted with staff trainers at Bizlink, who were trained to assist in the implementation of REAP with the participants. This was done to examine their perceptions of the usefulness of REAP as an adjunctive skills training to the center’s work therapy.

### REAP: Gamified augmented reality vocational training

The principles of Neuropsychological and Educational Approach to Remediation (NEAR) were used to develop the REAP program, with its emphasis on personalization, contextualization and transfer of learning to daily activities (24). The AR gamified training scenarios were developed using Unity 3D (25) and implemented on a RhinoX AR headset from Ximmerse (18). A set of handheld controllers came with the headset.

#### Gamified augmented reality platform

Four training scenarios were developed to contextualize the vocational training within a virtual sandwich café, namely (1) work etiquette, (2) sandwich making, (3) serving drinks, and (4) cashiering (18). The storyboard for each training scenario was developed based on the framework of Perceive, Recall, Plan and Perform system of task analysis, to break down the work tasks into steps to scaffold learning (22, 23). Participants could also personalize the game by typing in their names and assuming the role of an employee in the café.

In the “Work Etiquette” scenario, participants had to remember their work schedules and pick out the appropriate dress code, thereby training their attendance, timeliness and grooming. The working days were randomized into seven different permutations to reduce practice bias, while the clothing selections were also placed on random buttons. The system would generate visual hints upon unsuccessful attempts, such as “Tuesday is not a working day.” This enabled participants to acquire the ability to respond to verbal cues and prompts.

**FIGURE 1:**
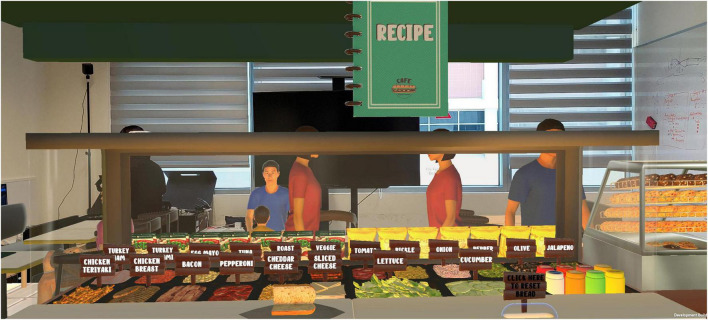
Sandwich making scenario.

In the “Sandwich Making” scenario, participants were given a tutorial on building a sandwich, after which they had to fulfill customers’ orders of different sandwich combinations. The participants would use the handheld controller to aim the laser pointer at the correct ingredient, then click and hold a button on the controller to pick up the item. A recipe book was available to provide information on the ingredients required for each sandwich, out of an array of 20 ingredients. Participants could flip-through the recipe book before it closed after 15 s of inactivity. Difficulty of this game increased with the increased number of ingredients and reduced visual cues on the ingredients required. For example, at levels one and two, participants were guided by a pointer to the right ingredients for the sandwiches. At level three, participants had to build sandwiches comprising three ingredients, but a message would pop up if they picked the wrong ingredients. At level four, the participants had to build sandwiches comprising up to five ingredients and there were no visual cues to alert them if they picked the wrong ingredients. Therefore, this scenario targeted workplace tolerance, instruction-taking, sustained attention and working memory. See Figure 1 for a screenshot of this scenario.

**FIGURE 2:**
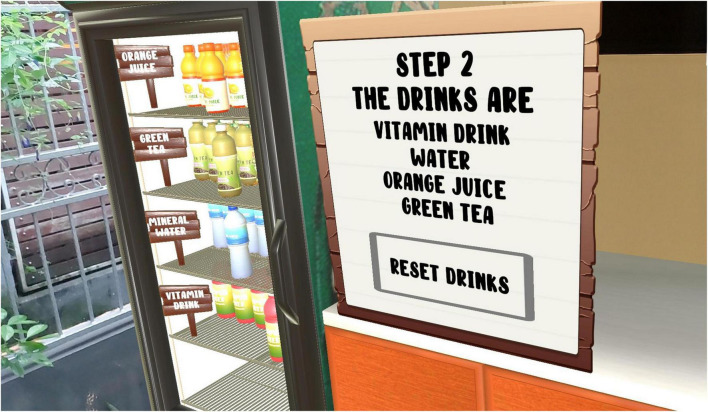
Serving drinks scenario.

In the “Serving Drinks” scenario, participants were required to pick up the correct drink from the refrigerator and place it on a serving tray. It involved in-hand manipulation of the handheld controller and some level of eye-hand coordination, as the participants were required to hold onto the controller’s button as they moved the drink and placed it on the allocated spot on the tray (see Figure 2). This scenario targeted adherence to workplace safety rules and use of proper body mechanics.

**FIGURE 3:**
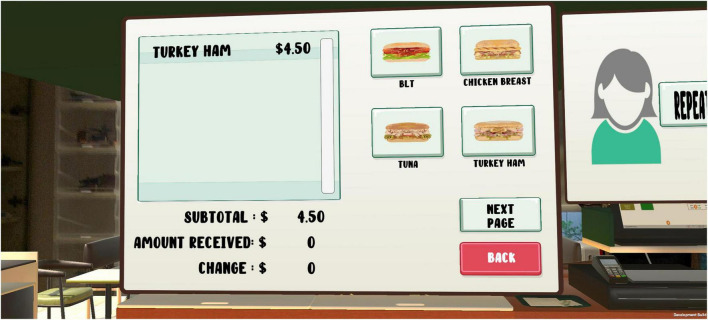
Cashiering scenario.

Lastly, the “Cashiering” scenario required participants to perform a series of steps on monetary transactions with the customers (see Figure 3). Participants were first prompted to greet the customers based on the time of day, followed by keying in the customers’ verbal orders into a point-of-sale machine. Upon receiving payment from customers, they would have to give the correct amount of change. Game difficulty was determined by the number of orders given by the customers and the number of times that the customers were asked to repeat the orders. For example, at level three, participants had to remember up to three order items (combinations of drinks and sandwiches) and they could click on the “repeat order” button up to three times to listen to the order again. At level four, participants had to remember up to five order items, which were also combinations of sandwiches and drinks. Besides working memory and money management skills, the scenario also targeted customer service.

During the development phase of the prototype, user testing was carried out with volunteers who consented and provided some preliminary feedback about the games, so that modifications could be made to enhance user acceptability. For more details of the AR gamified platform, please refer to Chiam et al. (18).

The AR system also collected data of the participants’ game performance. Such data included the duration of each game session, number of attempts made in the training scenarios during each session, whether the participants passed or failed each attempt and number of hints requested by the participants in each session. At the higher levels, the sandwich training scenario also included a few multiple-choice questions to test participants’ problem-solving and judgment in unexpected situations (for example, if an ingredient of a sandwich ran out). Number of errors of these multiple-choice questions per session was also captured.

#### REAP training protocol

During baseline assessments, participants were screened using the Neurobehavioral Cognitive Status Exam (Cognistat) (26) and the occupational therapists would then allocate different modules in the AR games according to participants’ cognitive functioning and vocational goals. A training plan was drawn up, where participants would either move across different scenarios of the same difficulty level, or increase the difficulty levels of each scenario. The number of prompts and cues given would also be tailored to each participant’s goals, functioning level and progress. In general, the participants were also allocated to “Beginner,” “Intermediate,” or “Advanced” level. At the “Beginner” level, participants could make use of the external cues within the gamified platform to complete each scenario. They could progress to the next difficulty level of each scenario when they accumulated ten correct attempts. At the “Intermediate” level, the participants were encouraged to utilize internal strategies as much as they could, instead of relying on external cues from the gamified platform. They could progress to the next difficulty level of each scenario when they accumulated ten correct attempts. At the “Advanced” level, the participants had to achieve ten consecutive correct attempts in each scenario without the use of external cues before they could progress to the next difficulty level. A set of remediation and compensatory strategies was also prepared, and the centers’ trainers were trained to assist in implementing these strategies according to the agreed plans and goals.

Participants participated in the REAP program three times a week for 10 weeks. In each week, two sessions involved AR games and one session was a bridging group. Each session lasted for 30 min. During the AR game session, participants engaged in the AR games and were given a break after every 10 min or as and when needed. Such frequency and duration of intervention were similar to most cognitive remediation programs (27) and within the duration range of programs that utilized virtual/augmented reality for persons with intellectual disabilities (5, 28). The participants engaged in different training scenarios as recommended and their performance was recorded by the trainers. Participants moved up the levels or across different training scenarios once they made ten successful attempts. Depending on their set goals, participants would be required to make ten consecutive successful attempts or non-consecutive ones. The usage of in-game visual and/or auditory hints also varied in accordance with the participants’ goals. In addition, cognitive strategies were taught during the games and reinforced during the bridging groups.

Bridging groups were designed to reinforce strategies learned during AR game sessions as well as to generalize the skills learned to real-life situations. These group sessions were facilitated by the research team, following an intervention manual. Through engagement in a variety of group games and activities, participants practiced their life skills and shared their AR game experiences with others. At the end of each bridging group, participants were reminded to use these cognitive strategies during their subsequent AR sessions.

### Outcome measures

The following assessments were carried out:

#### Vocational skills assessment: Feasibility evaluation checklist

The Feasibility Evaluation Checklist (FEC) is an observer-rated tool on work skills and behaviors that are relevant for persons with cognitive or physical disabilities (29). It measures aspects of general productivity, safety in the workplace and interpersonal behavior (29, 30). The rater will evaluate the person’s level of feasibility for competitive employment for each item, which is rated as “independent,” “minimal assist,” “moderate assist,” “not evaluated,” or “non-employable.” A total score is then computed, with a higher score indicating better vocational skills (30). FEC was administered by the trainer who was in-charge of the specific participant for work therapy. It was completed at baseline, post-training and eight-weeks after completion of the training. The participant might have a different trainer for the AR sessions, depending on the training schedule.

#### Cognitive assessment: Neurobehavioral cognitive status exam

Cognistat is a cognitive screening instrument widely used with adults with cognitive impairment (31, 32). The cognitive domains measured are: orientation, attention, language (comprehension, repetition and naming), constructional ability, verbal memory, mental calculations, and reasoning (similarities and judgment). Each domain (except for memory and orientation) has a screening component. If the participant fails the screening component, the metric component will be administered. A higher score indicates a higher level of cognitive performance in each domain (32). This assessment was carried out as part of a larger study involving the use of REAP with persons with neurodevelopmental and psychiatric disorders. Cognistat was administered at baseline, post-training and eight-weeks after completion of the training.

#### User feedback semi-structured interview

A semi-structured user feedback interview was also administered post-training with the participants, to obtain information on their experiences in participating in the REAP program. Questions were rephrased or simplified according to the level of understanding of the participants and the areas covered were:

1. The extent that participants found the REAP training program to be a useful component of their work therapy. Participants were asked to rate on a four-point Likert scale (1 = not useful at all to 4 = very useful). They were also asked for their opinions on the useful and non-useful aspects of the program.
2. Areas not covered in their standard work therapy that the REAP training program had addressed.
3. Participants’ interest in the REAP training (AR games and bridging groups): participants were asked to rate from 1 = not interesting at all to 4 = very interesting.
4. Ease of use: participants were asked to rate on a four-point Likert scale on whether the games were easy to understand (1 = not easy at all to 4 = very easy) and how comfortable it was for them to use the AR headset and hand controllers (1 = not comfortable at all to 4 = very comfortable). They were also asked to elaborate on their experiences.
5. Duration of the REAP program: participants were asked to rate on a three-point Likert scale (1 = too short, 2 = just right, 3 = too long).
6. How safe they felt when engaging in the AR games.
7. Overall experience of the program.

In addition, the user feedback semi-structured interview was conducted with trainers who assisted in implementing REAP with the participants. Questions covered the following aspects:

1. Trainers’ perception of the usefulness of REAP as an adjunct to the center’s work therapy: they were asked to rate on a four-point Likert scale (1 = not useful at all to 4 = very useful). Trainers were also asked for their opinions on the useful and non-useful aspects of the program.
2. Areas not covered in standard work therapy that the REAP program could uniquely address.
3. Strategies that trainers had utilized during the REAP program, which were generalized to daily activities and work therapy.
4. Ease of teaching participants to play the AR games: trainers were asked to rate on a four-point Likert scale on the level of ease in teaching the participants to play the games (1 = not easy at all to 4 = very easy). They were also asked to share their experiences in teaching the participants.
5. Duration of the REAP program: trainers were asked to rate on a three-point Likert scale (1 = too short, 2 = just right, 3 = too long).
6. Observed interest level of the participants: trainers were asked to rate from 1 = not interesting at all to 4 = very interesting.
7. Any safety concerns.
8. Feasibility of implementing the REAP program across more sites: trainers were asked to rate from 1 = not feasible at all to 4 = feasible for all the sites.
9. Overall experience of the program.

Quantitative and qualitative data were obtained from these semi-structured interviews.

### Study procedure

Recruitment was conducted at Bizlink Center’s Headquarters and the Day Activity Center. Purposive sampling was done by the center managers and staff, based on the inclusion/exclusion criteria and clients’ goal of engaging in work-related training. The study was explained to the clients and caregivers and consent was taken before commencement of the study. Ethics approval was obtained from the Singapore Institute of Technology Institutional Review Board (study number: 2020004). All participants and their caregivers consented to the participation of this study before REAP training was implemented.

Upon informed consent, the following procedures were implemented:

1. Baseline assessments: (within the two weeks before commencement of REAP training): Feasibility Evaluation Checklist (FEC) and Cognistat were administered. The Cognistat was administered by the research team with the participants, while FEC was administered by the trainers.
2. REAP training program: research team worked with the centers’ trainers to conduct the AR games and bridging groups. Sessions were conducted three times a week for 10 weeks (30 sessions).
3. Post-training assessments (within the two weeks after completion of REAP training): FEC and Cognistat were administered. The research team also carried out the user feedback interviews with the participants and trainers.
4. Eight-week follow-up assessments: FEC and Cognistat were again administered to evaluate any change in vocational skills/behavior and cognitive functioning.

### Data analyses

Quantitative statistical analyses were performed using IBM SPSS Statistics Version 28 (33). Descriptive statistical data was obtained from the participants’ demographic profiles, user feedback interviews and game performance (duration per session, number of correct attempts, number of in-game hints, number of errors in the multiple-choice questions, etc.). Repeated Measures ANOVA was used to test for changes in vocational skills and cognitive functioning as measured by FEC and Cognistat, respectively. Repeated Measures ANOVA was also conducted to test for changes in game performance over the beginning, middle and final sessions. Statistical significance was set at *p* ≤ 0.05.

The user feedback qualitative data was entered into Quirkos 2.4.2 (34) and thematic analysis was carried out. The data was labeled and coded by the first author and a codebook was created to describe each code. The first, third, and fourth authors looked through the codes to determine convergence and divergence through an iterative process, as well as to make comparisons between participants’ and trainers’ experiences (35).

## Results

### Participants’ demographics

A total of 15 participants and 11 trainers took part in this pilot study. The participants’ mean age was 31.47 years (SD = 12.07) and 53.30% of them were females. 11 participants had intellectual disabilities while four participants had autism spectrum disorder. All participants were unmarried at the point of study. Other demographic profiles are shown in Table 1.

**TABLE 1:** Participants’ demographic profile and game level.

Profile and game level (*n* = 15)	*N* (%)
**Gender**	
Female	8 (53.3%)
Male	7 (46.7%)
**Setting**	
Bizlink Headquarters	9 (60.0%)
Bizlink day activity center	6 (40.0%)
**Comorbid psychiatric conditions**	
None	10 (66.7%)
Depression	3 (20.00%)
Schizophrenia	1 (6.7%)
Attention-deficit hyperactivity disorder	1 (6.70%)
**Years of receiving services at Bizlink center**	
0–5 years	10 (66.7%)
6–10 years	4 (26.7%)
11 years and above	1 (6.7%)
**Initial game level**	
Beginner	13 (86.7%)
Intermediate	2 (13.3%)
**Final game level achieved**	
Beginner	10 (66.7%)
Intermediate	1 (6.7%)
Advanced	4 (26.7%)
**Participants’ profile (*n* = 15)**	***Mean (SD)***
Age (in years)	31.47 (12.07)
Years of education	14.82 (1.93)

### Game performance

Based on initial cognitive screening, 13 participants were allocated to the Beginner game level and two were allocated to the Intermediate level. At the end of the 10-week program, ten participants remained at the Beginner level, one went up to the Intermediate level and four participants achieved the Advanced level (see Table 1). Participants who continued to require external cues from the gamified platform would stay in the Beginner level, while the participant who did not rely on external cues to get correct responses was at the Intermediate level. Finally, participants who were able to achieve ten consecutive correct attempts moved up to the Advanced level.

Repeated Measures ANOVA was conducted to test for changes in game performance over the beginning, middle and final sessions. As Mauchly’s Test indicated that the assumption of sphericity had been violated for the measurements of “duration per session” and “number of attempts made per session,” χ^2^ (2) = 6.16–9.96, *p* < 0.05, degrees of freedom were corrected using Greenhouse-Geisser estimates of sphericity (ε = 0.65–0.73). Results showed that there were no significant time effects on these two measures. On the other hand, Mauchly’s Test showed that the assumption of sphericity had been met for the other game performance measurements. With sphericity assumed, it was found that there were significant reduction in number of wrong attempts in the multiple-choice questions over the three time-points *F*(2,28) = 4.52, *p* = 0.02, η*p*^2^ = 0.24. Table 2 shows the mean scores and the time effect of the game performance measurements over the three time points.

**TABLE 2:** Repeated measures for mean scores on augmented reality game performance at beginning, middle, and final sessions.

Game measurement	Beginning session Mean (SD)	Middle session Mean (SD)	Final session Mean (SD)	Time effect			
				F	*df*	*p*	η *p*^2^
Duration per session (minutes)	27.13 (18.30)	26.87 (7.59)	24.60 (6.10)	0.21	1.30	0.72	0.02
Number of attempts per session	7.67 (4.88)	9.80 (7.06)	9.07 (7.26)	0.45	1.45	0.58	0.03
Number of correct attempts	6.13 (4.42)	8.40 (7.03)	7.88 (6.85)	21.07	2	0.56	0.04
Number of wrong attempts	1.53 (1.41)	1.40 (1.81)	1.33 (1.45)	0.68	2	0.93	0.01
Number of hints requested	0.80 (1.32)	3.00 (4.97)	2.73 (4.03)	1.94	2	0.16	0.12
Number of errors in multiple-choice questions	3.67 (4.25)	1.00 (2.65)	0.80 (1.70)	4.52	2	*0.02	0.24

*Significant at p ≤ 0.05.

### Vocational skills and cognitive skills

Repeated Measures ANOVA was also carried out to investigate changes in vocational and cognitive skills as measured by FEC and Cognistat, respectively. FEC and Cognistat scores were obtained at baseline, post-training and eight weeks after training. One participant became unwell just before Cognistat was due for administration at the eight-week follow-up period. As a result, full data could not be obtained from her and her Cognistat scores were not used for the data analysis.

Mauchly’s Test indicated that the assumption of sphericity had been violated for the Cognistat domains of “orientation” and “similarity,” χ^2^ (2) = 7.63–12.39, *p* < 0.05. Therefore, degrees of freedom were corrected using Greenhouse-Geisser estimates of sphericity (ε = 0.61–0.68). Results showed that there were no significant time effects on these two cognitive domains. On the other hand, Mauchly’s Test showed that the assumption of sphericity had been met for the measurements of FEC total scores, Cognistat total scores and all the other Cognistat domain scores. With sphericity assumed, it was found that there were significant improvements in FEC total scores, Cognistat memory domain, Cognistat reasoning (judgment) domain and Cognistat total scores over the three time-points *F*(2,26–28) = 3.29–4.76, *p* < 0.05, η*p*^2^ = 0.20–0.27. Table 3 shows the mean scores and the time effect of the vocational and cognitive skills measurements over the three time points.

**TABLE 3:** Repeated measures for Feasibility Evaluation Checklist (FEC) total scores and Cognistat domain and total scores at baseline, post-training, and at eight-week follow-up.

Measurement	Baseline Mean (SD)	Post-intervention Mean (SD)	Eight-week Follow-up Mean (SD)	Time effect			
				F	*df*	*p*	η*p*^2^
**Feasibility Evaluation Checklist (FEC) total**	42.40 (11.53)	47.07 (12.82)	49.73 (16.34)	3.74	2	*0.04	0.21
Cognistat orientation	9.64 (3.54)	10.50 (2.68)	9.07 (3.08)	1.17	1.36	0.31	0.08
Cognistat attention	4.79 (2.64)	6.00 (2.96)	5.93 (2.34)	1.45	2	0.25	0.10
Cognistat comprehension	3.79 (1.63)	4.36 (1.15)	4.36 (1.69)	2.25	2	0.13	0.15
Cognistat repetition	6.64 (3.37)	7.43 (3.11)	7.86 (3.30)	0.90	1.80	0.41	0.07
Cognistat naming	5.71 (1.44)	5.71 (1.07)	6.00 (1.11)	1.07	2	0.36	0.08
Cognistat construction ability	3.71 (1.98)	3.57 (1.28)	3.36 (1.91)	0.38	2	0.69	0.03
Cognistat memory	3.57 (3.86)	7.36 (4.55)	7.50 (4.67)	10.21	2	* <0.001	0.44
Cognistat calculations	2.36 (2.06)	3.07 (1.44)	2.50 (1.35)	1.63	2	0.22	0.11
Cognistat Similarities	1.64 (2.53)	2.57 (3.18)	3.21 (3.47)	3.28	1.22	0.08	0.20
Cognistat judgment	0.86 (1.51)	1.43 (1.34)	2.14 (1.99)	4.76	2	*0.02	0.27
Cognistat total	42.71 (17.18)	52.00 (14.79)	51.50 (18.49)	4.53	2	*0.02	0.26

*Significant at p ≤ 0.05.

### Participants and trainers’ feedback-quantitative results

**FIGURE 4:**
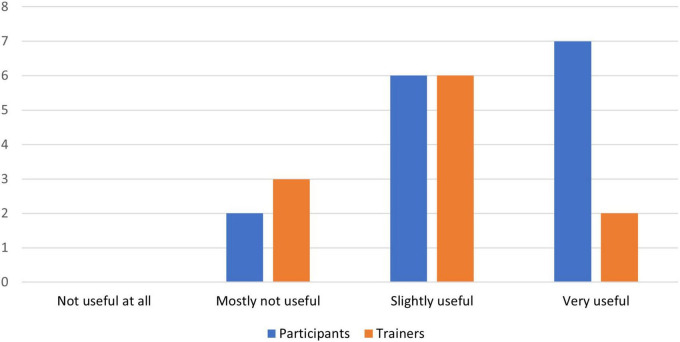
Feedback on usefulness of the Augmented Reality Games to Enhance Vocational Ability of Patients (REAP) program.

**FIGURE 5:**
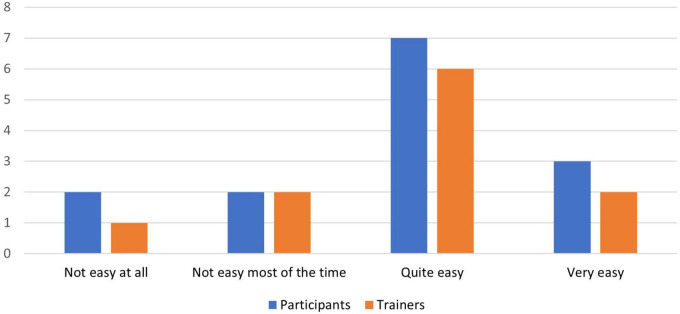
Feedback on the ease of use of the Augmented Reality Games to Enhance Vocational Ability of Patients (REAP) augmented reality games.

**FIGURE 6:**
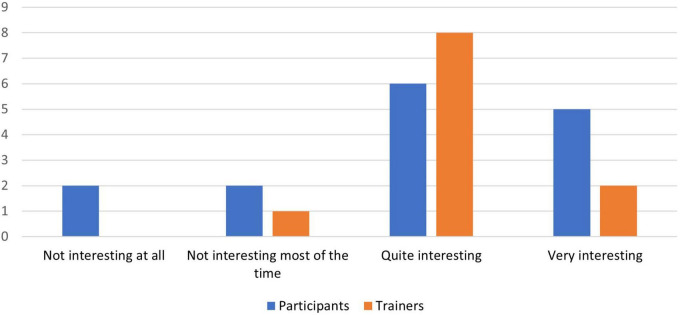
Feedback on the interest level of the Augmented Reality Games to Enhance Vocational Ability of Patients (REAP) program.

**FIGURE 7:**
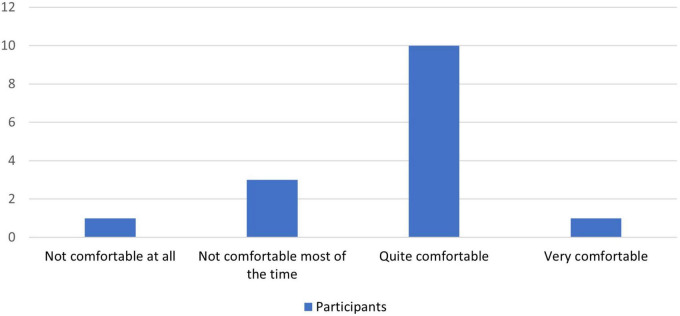
Feedback on the comfort level of using the Augmented Reality Games to Enhance Vocational Ability of Patients (REAP) augmented reality equipment.

**FIGURE 8:**
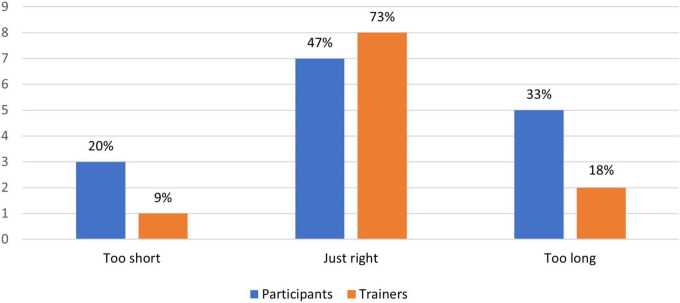
Feedback on the duration of the Augmented Reality Games to Enhance Vocational Ability of Patients (REAP) program.

Figures 4–8 show the user ratings of participants and trainers on the usefulness, ease of use, interest, comfort level and duration of the REAP program. As seen from the figures, majority of the participants and trainers found the program to be slightly useful or very useful. While most of them found the AR games to be easy to understand and interesting, a small number of them had some difficulty with it. The participants also largely found the AR equipment to be quite comfortable or very comfortable to use. In terms of the duration of the REAP program, about 47% of the participants found the duration to be just right, with 20% finding it too short and 33% finding it too long. Conversely, 73% of the trainers found the duration to be just right, while 9% found the program to be too short and 18% found it to be too long.

### Participants and trainers’ feedback-qualitative results

Four themes were generated from the thematic analysis of the interviews with participants and trainers. They were: (1) benefits of games and technology; (2) skills training and strategy learning; (3) tailoring the program to meet individual needs and contexts; and (4) technical aspects of the augmented reality platform. Participants’ quotes were labeled with “P,” while trainers’ quotes were labeled with “T.”

#### Benefits of games and technology

Six trainers felt that the nature of augmented reality provided a unique experience for participants and staff to experience working in a food and beverage industry, while picking up a new technological skill. It was also deemed to be less costly because there was no need to set up a real café and virtual ingredients could be “reused.” Nine participants indicated that the program enabled them to try out new things in a virtual world, which were different from their standard vocational rehabilitation. They could experiment with behaviors associated with tasks such as taking customers’ orders and cashiering in a self-paced and less anxiety provoking manner:

*“Can learn things in a different way*… *and how to manage situation that is happening and how to break things into parts. Eg what is most important, what is less important, what is not important. Those that are not important can slowly take my time to figure out” (P16).*

This was echoed by trainers, who stated that many of their clients might not get a chance to interact with customers or do cashiering and they could “*learn better when they could try things out through game*” (T04).

Five participants and two trainers also highlighted that the program was fun and engaging. Some of them found it to be an interesting and relaxing way to learn and became more motivated:

*“Fun, exciting for clients, it is a good bonding time for clients and trainers. It can increase sense of confidence for some clients and allow them to learn new things” (T05).*

One trainer also pointed out that the use of augmented reality platform minimized direct physical contact with participants during COVID-19 pandemic period, which helped in the execution of training.

#### Skills training and strategy learning

Seven participants and four trainers felt that the program trained aspects of cognitive functions, such as attention, memory and problem-solving. Memory recall was specifically identified by participants as an area that they benefited in. When asked about cognitive strategies used during the games, the trainers identified breaking down the tasks, verbalizing the ingredients aloud, using visual aids and giving verbal prompts as the commonly used ones.

Majority of the trainers also felt that the program taught new skills such as work etiquette, customer service, reading and use of technology. Conversely, only three participants stated that they picked up new skills during the process, which were mainly soft skills and self-regulation skills:

*“Learn how to be patient when you start to be stressed out and it’s difficult to calm down and be patient. I’m a person who gets angry easily, Have to tell myself to calm down and do things step by step in front of me” (P16).*

Participants and trainers also feedback that the psychomotor skills required in the “Serving Drinks” scenario were too demanding, as it entailed precise manipulation of the controller to point at the objects accurately.

In addition, both groups gave comments on how to maximize transfer of learning to real-life situations and provided suggestions for other virtual scenarios. They felt that opportunities should be given to try out customer service, remembering things, doing chores and food preparation in the daily lives, so as to maintain the skills learned. The participants and trainers also suggested other virtual scenarios such as packing items, data entry and housekeeping, so as to closely mimic participants’ work experiences or to expand their repertoire of skills.

#### Tailoring the program to meet individual needs and contexts

The participants and trainers provided a range of comments regarding the difficulty level of different scenarios. Some participants felt that the “Sandwich Making” scenario was easy and the “Cashiering” scenario challenging, while others had the opposite experiences. Likewise, a few trainers felt that rehearsal strategies helped participants in remembering customers’ orders at the cashier better, while other trainers observed this to be a frustrating experience for the participants.

In terms of perceived usefulness of the gamified platform, there were also differing opinions among the participants, as well as between participants and trainers. Some of the participants found the AR games more useful and interesting from the middle sessions onward, while others found the initial sessions more beneficial for them. Three trainers found the “Work Etiquette” scenario particularly useful, four trainers found the “Cashiering” scenario to be particularly useful, while four participants found the “Sandwich Making” scenario to be particularly useful. It appeared that the program catered to differing needs and interest levels and provided a good mix of challenges. Therefore, six trainers emphasized the need to tailor the program according to individual needs, through adjusting the duration of each session, number of sessions, number of cues and prompts, etc. They also felt that participants with lower cognitive functioning and poorer reading levels tend to struggle with the auditory and written instructions. Therefore, instructions would need to be presented slower, with more visual cues in the virtual environment to direct their attention appropriately.

*“For participants with harder understanding: recommend guiding one time round in game as clients are more visual. Suggest at level 1, to show sandwich already prepared in shelf” (T06).*

In addition, trainers who were more involved in sheltered work found this program to be less applicable, thus highlighting the importance of contextualizing the program.

#### Technical aspects of the augmented reality platform

Four trainers and two participants highlighted technical aspects of the AR platform which could be improved, such as software bugs, sensitivity of the handheld control and inconsistent audio feedback. They also suggested the need to have a working second-player mode installed on the mobile device, for trainers to view the participants’ engagement in the games concurrently. This would allow the trainers to monitor the participants’ progress and to provide appropriate prompts more easily.

*“It was difficult to see what is on the screen, especially when clients cannot find the laser pointer, so good to have two screens so that trainer knows what client is viewing on the screen and can help” (T02).*

In addition, eight participants and five trainers commented on the comfort and safety aspects of the AR platform. Although a few of them felt that the headset was comfortable enough, others found it too heavy after some time. Therefore, it was important to have a break every 10 min to reduce discomfort and prevent giddiness. Proper adjustment of the headset was also crucial to ensure that it was not too tight. In addition, three trainers stressed the need to have adequate space around the participants, to accommodate physical movements when the participants were navigating around the virtual environment. Lastly, five trainers commented that the use of an AR headset rendered it unsuitable to be used as a home program. A mobile application version would be required.

## Discussion

This pilot study showed that adults with intellectual and developmental disabilities generally had positive experiences with a vocational training program that utilized a gamified augmented reality platform, coupled with intervention sessions to bridge real-life applications. In addition, participants who had engaged in this 10-week program showed a significant improvement in vocational skills and aspects of cognitive skills, which were maintained eight weeks after training. Vocational skills and cognitive skills were measured using Feasibility Evaluation Checklist (FEC) and Cognistat, respectively.

### Acceptability of augmented reality vocational training program

Research on the user acceptability of AR gamified vocational training programs for adults with intellectual and developmental disabilities has been sparse. In the small-sample study on the AR-enabled vocational task prompting system (ARCoach), the three research participants indicated that the mental and physical effort in operating the device was low and that they would recommend it to their peers (6). Similarly, 85.7% of the participants who used the “Paint-cAR” mobile application device to learn car paint repair also stated that they would like to use AR-enabled mobile applications to learn more vocational skills (14). Nevertheless, 50% of these participants reported that they hardly installed new mobile applications and would need technical support (14). In another study, adults with intellectual disabilities working in horticulture found it difficult to activate AR video instructions at selected locations due to navigation difficulties and they did not find the technology useful (13).

Our pilot study provided a more in-depth understanding of the feasibility and acceptability of gamified AR programs by conducting semi-structured interviews with both participants and vocational rehabilitation trainers. The AR platform used in the REAP program was displayed on a headset rather than a mobile device application. This provided a more immersive environment without the need for location-specific triggers. However, participants commented on the comfort level of the headsets, with some finding it heavy after a while and emphasized the need for proper adjustments of the headsets and to have frequent breaks to prevent giddiness. In general, majority of the participants and trainers found the REAP program to be useful and interesting. They also found the AR games to be user-friendly and the equipment to be relatively easy to handle.

### Effectiveness of the augmented reality vocational training program

The qualitative data provided further insights into the acceptability and effectiveness of the REAP program. Seven participants and four trainers felt that the program trained aspects of cognitive functions, such as attention, memory, and problem-solving. This was reflected in their improvement in the memory and reasoning domain scores of the Cognistat. Although adults with intellectual disabilities had cognitive limitations, the use of a contextualized skills training program that promoted rehearsal and simple strategy building appeared to have helped these participants in performing cognitive tasks. Memory recall was specifically identified by participants as an area that they benefited in, as scenarios such as “Sandwich Making” and “Cashiering” required them to find ways to memorize sandwich ingredients or multiple orders from customers. When asked about cognitive strategies used during the game sessions, the trainers identified breaking down the tasks, verbalizing the ingredients aloud, using visual aids and giving verbal prompts as the commonly used strategies. Some of these strategies might have facilitated internal strategy learning process, while others might be compensatory in nature. Therefore, the participants could have picked up these strategies to varying degrees and applied them during the game sessions and across various tasks during bridging sessions. In the field of cognitive remediation within psychiatric rehabilitation, the use of strategies had also been shown to have a positive effect on functioning (36, 37). More research would be necessary to ascertain the strategy learning process of adults with intellectual and developmental disabilities.

In addition, the participants commented that they picked up soft skills and self-regulation skills during the REAP training program. As self-management skills could have an impact on work task performance (38), improvement in such skills could have enhanced the participants’ interpersonal behavior and work productivity, which were components evaluated in the FEC. Correspondingly, the trainers reported that the REAP program taught participants new skills such as work etiquette, customer service and instruction-taking. Although participants might not be serving customers in the center, the AR games had provided opportunities for them to learn workplace interaction, executing work instructions and correct work habits in the virtual work environment. This could have led to an improvement in their total FEC scores, which were maintained eight weeks after the REAP program ended. Therefore, there is potential in using a well-integrated AR-enabled program to improve functional performance of adults with intellectual and developmental conditions.

A notable theme that emerged from the interviews was the importance of tailoring the program according to individual needs and contexts. There were differing opinions from participants and trainers about the useful aspects of the training scenarios, and they also had different perceptions of the scenarios’ difficulty levels. It appeared that the REAP program catered to differing needs and interest levels and provided a good mix of challenges. Therefore, it would be beneficial for an AR-enabled vocational training program to offer a range of work tasks that are structured in various difficulty levels and to provide an array of visual and auditory cues. The vocational training staff will then have to conduct a functional assessment to determine the type of virtual work scenarios, the difficulty level, the type of cues and the intensity of sessions that best match the client’s functional level and vocational goals.

From the results of the participants’ game performance, it was found that there were no significant changes in the number of attempts per session, number correct attempts and the number of hints used across the sessions. Perhaps as the participants moved along the sessions, they attempted different training scenarios or moved up the difficulty levels. Therefore, they might not necessarily have accomplished more attempts or had more correct attempts. However, there were significant improvement in the number of correct multiple-choice responses, showing that some learning had taken place. There is currently no evidence on the minimal number of sessions required for an AR-enabled vocational training program for adults with intellectual and developmental conditions. In this study, a 10-week program was able to bring about some gains in vocational skills. More research would be needed to investigate the intensity required for persons of different levels of intellectual disabilities with diverse vocational goals.

The participants and trainers highlighted that the AR training scenarios provided a unique experience and gave opportunities to try things out in a virtual world, before implementing them in the real world. Through these virtual games, the trainers got to understand the type of cues and supports (e.g., written instructions, visual arrows, auditory prompts, flipping through the recipe book, etc.) that were effective with specific participants and were able to implement these cues in their standard work therapy within the center. During the COVID-19 pandemic when physical contact was restricted, AR games also provided an alternative source of work task engagement. Hence, AR may be a viable option for adults with intellectual disabilities to try out different “virtual jobs,” to understand their job preferences and interests. Despite supported or open employment being the preferred employment model for adults with intellectual and developmental disabilities, many of them are still not able to access such employment opportunities and may only be able to attend sheltered workshops (39). Through exposure to various “virtual jobs,” vocational staff can start to identify clients’ strengths, interests and preferences and find similar vocational opportunities in the job market for clients to embark on.

### Study limitations and recommendations for future research

This pilot study adopted a pretest–posttest mixed methods design, with a small sample size and no comparison group. An experimental study would be necessary to determine the effectiveness of a gamified augmented reality vocational training program, when compared against standard vocational rehabilitation. As this study recruited adults with intellectual disabilities and autism who also had co-morbid psychiatric conditions, the participants reported varying opinions on the useful aspects of the training scenarios. Future studies would be needed to have a thorough understanding of the therapeutic components that matched different adaptive functioning levels of this population of clients. Comparisons in responses between participants with intellectual disabilities and autism will also shed some light on the therapeutic ingredients of this program.

In addition, it would be useful to delve deeper into how therapeutic alliance and curriculum of the bridging group could affect training outcomes. As the trainers were working closely with the participants in the work therapy program within the center, they were assigned to administer the FEC. Future studies involving blinding of the raters would increase the internal validity of the research.

Literature on supported employment has shown that persons with intellectual disabilities not only experience barriers in obtaining employment, but also have difficulties in maintaining employment (40, 41). The use of AR-enabled vocational training for clients on supported employment has not been explored, but there is potential for its use as an adjunctive training to improve job sustainability.

Lastly, participants and trainers in this study reported technical issues on the AR platform and suggested enhancements such as using google glasses and having a second player mode. In addition, it was acknowledged that the hand grip movements used in manipulating the handheld controllers were not similar to the hand grips and pinches used in the real-life functional tasks of sandwich making and serving drinks. Therefore, a more realistic physical interaction with virtual objects would have improved user experience. A wider variety of virtual jobs and training scenarios could also have improved user satisfaction and increased its effectiveness. Future research could explore a wider repertoire of AR gamified vocational training scenarios, with additional features such as verbal interactions and haptic feedback.

## Conclusion

This pilot study on the REAP program showed that a gamified augmented reality vocational training program was feasible and accepted by both adults with intellectual and developmental disabilities, as well as their vocational rehabilitation trainers. When integrated with bridging sessions to facilitate transfer of learning to existing work therapy, participants on the REAP program showed significant improvements in vocational skills and aspects of cognitive performance which were maintained eight weeks after the program ended. Future experimental studies with larger sample size could provide stronger evidence on its effectiveness in improving vocational outcomes.

## Data availability statement

The original contributions presented in this study are included in the article/Supplementary material, further inquiries can be directed to the corresponding author.

## Ethics statement

The studies involving human participants were reviewed and approved by Singapore Institute of Technology Institutional Review Board (study number: 2020004). The participants provided their written informed consent to participate in this study.

## Author contributions

B-LT, FG, and AM: conception and design. B-LT, IL, SK, OD, and FG: development of REAP vocational training program. B-LT, IL, and SK: data analysis. All authors wrote the manuscript and approved the submitted version.

## Supplementary material

Supplementary Datasheet 1 User Feedback Semi-Structured Interview Form for Participants.

Supplementary Datasheet 2 User Feedback Semi-Structured Interview Form for Vocational Trainers.

## Abbreviations

AR: Augmented Reality
REAP: Augmented Reality Games to Enhance Vocational Ability of Patients
NEAR: Neuropsychological and Educational Approach to Remediation.
