# Supplementary material for: A gamified augmented reality vocational training program for adults with intellectual and developmental disabilities: A pilot study on acceptability and effectiveness
Source: Front Psychiatry. 2022 Aug 4;13:966080. doi: 10.3389/fpsyt.2022.966080 (PMC9386351; doi:10.3389/fpsyt.2022.966080)
Supplement: Supplementary Datasheet 1 — User Feedback Semi-Structured Interview Form for Participants. [file Data_Sheet_1.PDF]

==

Participant's Semi-structured Interview on Augmented Reality Games to Enhance Vocational Ability of Patients (REAP)

To be completed after REAP. This is a semi-structured interview and will be conducted by the study administrator.

Usefulness

1) How do you rate the usefulness of the REAP programme in training your thinking skills and vocational skills (please circle as appropriate)?

   1= not useful at all
   2= mostly not useful
   3= slightly useful
   4= very useful

2) Which are the useful and not useful aspects?

____

____

3) If rated '3' and '4' in Question 1, which session did you begin to find the REAP games useful?

____

____

4) Which areas REAP can uniquely address, which are not addressed by standard vocational training?

____

____

==

Ease of use

5) How easy is it for you to understand how to play the REAP games? (please circle as appropriate)?:

1= not easy at all
2= not easy most of the time
3= quite easy
4= very easy

6) Which are the games that are easy to understand? Which are the games that are difficult?

____

____

7) How is the duration of engaging in REAP (15 minutes)?

1= Too short
2= Just right
3= Too long

8) How comfortable is it for you to use the REAP equipment (please circle as appropriate)?:

1= not comfortable at all
2= not comfortable most of the time
3= quite comfortable
4= very comfortable

9) Describe your experience of using the equipment and playing the games.

____

____

10) How interested are you in playing the REAP games? (Please circle as appropriate):

1= not interesting at all
2= not interesting most of the time
3= quite interesting
4= very interesting

11) Do you have any safety concerns about using the REAP games?

Yes/No. If Yes, please elaborate: ____

____

User Feedback Semi-Structured Interview (Participants)

==

12) Any other feedback about your experience using the REAP as part of your vocational training.

Date of completion:
Name and signature of administrator conducting the survey:
