# Supplementary material for: A gamified augmented reality vocational training program for adults with intellectual and developmental disabilities: A pilot study on acceptability and effectiveness
Source: Front Psychiatry. 2022 Aug 4;13:966080. doi: 10.3389/fpsyt.2022.966080 (PMC9386351; doi:10.3389/fpsyt.2022.966080)
Supplement: Supplementary Datasheet 2 — User Feedback Semi-Structured Interview Form for Vocational Trainers. [file Data_Sheet_2.PDF]

===

Vocational Staff Semi-structured Interview on Augmented Reality Games to Enhance Vocational Ability of Patients (REAP)

To be completed after REAP. This is a semi-structured interview and will be conducted by the study administrator.

Usefulness

1) How do you rate the usefulness of the REAP programme as an adjunct to vocational training. (please circle as appropriate)?

   1= not useful at all
   2= mostly not useful
   3= slightly useful
   4= very useful

2) Which are the useful and not useful aspects?

___

___

3) If rated '3' and '4' in Question 1, which sessions did you begin to find the REAP games useful for the service users?

___

___

4) Which areas REAP can uniquely address, which are not addressed by standard vocational training?

___

___

==

5) What are the cognitive strategies that you have utilised during REAP programme, which are generalised during vocational training?

____

____

Ease of use

6) How easy is it for you to teach service users how to play the REAP games? (please circle as appropriate)?:

1= not easy at all
2= not easy most of the time
3= quite easy
4= very easy

7) Which are the games that are easy to teach? Which are the games that are difficult?

____

____

8) How is the duration of engaging in REAP (15 minutes)?

1= Too short
2= Just right
3= Too long

9) How feasible will it be to use REAP across more vocational training sites?

1= not feasible at all
2= not feasible across most sites
3= Feasible for some sites
4= Feasible for all the sites.

10) How feasible is it to use REAP as a home programme?

1= not feasible at all
2= Feasible but with some modifications (please state)

____

3= Feasible to use

==

11) Describe your experience of using the REAP as an adjunct to your vocational training programme.

____

____

12) How interested are the service users in playing the REAP games? (please circle as appropriate)?:

1= not interesting at all
2= not interesting most of the time
3= quite interesting
4= very interesting

13) Do you have any safety concerns about using the REAP games?

Yes/No. If Yes, please elaborate: ____

____

14) Any other feedback about your experience using the REAP as an intervention programme.

____

____

____

____

____

Date of completion:
Name and signature of administrator conducting the survey:
